# Genome-Wide Association Study of Schizophrenia in Japanese Population

**DOI:** 10.1371/journal.pone.0020468

**Published:** 2011-06-06

**Authors:** Kazuo Yamada, Yoshimi Iwayama, Eiji Hattori, Kazuya Iwamoto, Tomoko Toyota, Tetsuo Ohnishi, Hisako Ohba, Motoko Maekawa, Tadafumi Kato, Takeo Yoshikawa

**Affiliations:** 1 Laboratory for Molecular Psychiatry, RIKEN Brain Science Institute, Saitama, Japan; 2 Laboratory for Molecular Dynamics of Mental Disorders, RIKEN Brain Science Institute, Saitama, Japan; Chiba University Center for Forensic Mental Health, Japan

## Abstract

Schizophrenia is a devastating neuropsychiatric disorder with genetically complex traits. Genetic variants should explain a considerable portion of the risk for schizophrenia, and genome-wide association study (GWAS) is a potentially powerful tool for identifying the risk variants that underlie the disease. Here, we report the results of a three-stage analysis of three independent cohorts consisting of a total of 2,535 samples from Japanese and Chinese populations for searching schizophrenia susceptibility genes using a GWAS approach. Firstly, we examined 115,770 single nucleotide polymorphisms (SNPs) in 120 patient-parents trio samples from Japanese schizophrenia pedigrees. In stage II, we evaluated 1,632 SNPs (1,159 SNPs of *p*<0.01 and 473 SNPs of *p*<0.05 that located in previously reported linkage regions). The second sample consisted of 1,012 case-control samples of Japanese origin. The most significant *p* value was obtained for the SNP in the *ELAVL2* [(embryonic lethal, abnormal vision, Drosophila)-like 2] gene located on 9p21.3 (*p* = 0.00087). In stage III, we scrutinized the *ELAVL2* gene by genotyping gene-centric tagSNPs in the third sample set of 293 family samples (1,163 individuals) of Chinese descent and the SNP in the gene showed a nominal association with schizophrenia in Chinese population (*p* = 0.026). The current data in Asian population would be helpful for deciphering ethnic diversity of schizophrenia etiology.

## Introduction

Schizophrenia is a debilitating mental disorder characterized by psychotic manifestations including hallucinations, delusions, and cognitive deficits. Despite the high heritability of the disease estimated at up to 80%, key molecules and/or molecular pathways underlying the disease are still elusive. Candidate gene-based analyses have an inherent limitation, that is, we do not know the precise pathophysiological basis for the disease. However, through the rapid development of genotyping technology, it has become feasible to genotype hundreds of thousands of single nucleotide polymorphisms (SNPs) covering the whole human genome. A shift toward genome-wide association study (GWAS) from a gene-based approach is accelerated. To date, a number of GWASs of psychiatric disorders, including schizophrenia, have been reported [1], [2], [3], [4], [5], [6], [7]. These have produced substantial evidence for the association of the disease with specific risk loci. For instance, O'Donovan et al. reported the evidence for association around *ZNF804A* (*p* = 1.61×10^−7^) [2]. However, the protein encoded by *ZNF804A* is uncharacterized and its function is unknown. No functional candidate genes that stemmed from current understanding of schizophrenia pathophysiology surpassed the genome-wide significance level in that study. It is also noteworthy that many GWASs so far have potentially missed the true association of the genes with small effect, because of a stringent threshold. Conversely, a liberal threshold requires follow-up studies to eliminate false positives from genuine associations. Therefore, a simple procedure for overcoming this problem is the use of a multistage screening approach, using a modest threshold in each stage. In addition, case-control design is liable to population stratification, which can cause spurious associations. To eliminate false positives due to population stratification and other confounding factors, the transmission disequilibrium test (TDT) design that uses patients and their parents (trios) is preferable as an alternative approach.

In this study, starting from a whole genome association survey of trio families, we carried out a staged association study for schizophrenia by analyzing three sets of samples, two from Japanese cohort and one from Chinese population, which is ethnically close to Japanese. All three sets of our samples showed a nominally significant association with a SNP on the *ELAVL2* gene.

This Asian GWAS of schizophrenia is hoped to provide a broader view of the genetic basis of schizophrenia, because schizophrenia GWASs to date are much accumulated in European descent.

## Results

### Stage I: GWAS of Japanese trio samples

Because of concerns regarding population stratification and other unknown confounding factors, we performed the first-stage screening restricted to pedigree trio samples comprising 120 families, each consisting of a patient with schizophrenia and their parents. All the subjects were Japanese and diagnosis of schizophrenia was carried out by at least two experienced psychiatrists according to DSM-IV criteria, on the basis of interview and medical records.

The trios were initially genotyped using Affymetrix GeneChip Mapping 100 K Arrays. Out of a total of 115,770 SNPs, 97,963 SNPs were successfully genotyped. The rest, 17,807 SNPs, were nonpolymorphic in the Japanese population or failed at the genotyping stage. They were excluded from further analyses. We ranked genotyped SNPs on the basis of strength of association using the allelic association test. Nominally significant results were detected for 1,159 SNPs (*p*<0.01).

**Figure 1:**
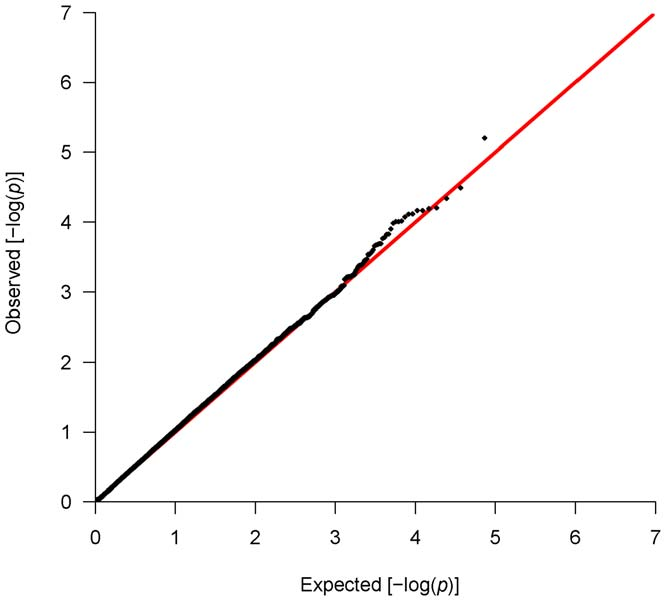
A quantile-quantile (QQ) plot for association results of the first-stage analysis. The empirical and theoretical distributions are shown as dots and line, respectively.

Genotyping data yielded an average call rate of 96.6%, and apparent inheritance errors in trio samples were detected in <0.2% of all SNPs. A quantile-quantile (QQ) plot for association results is provided in Figure 1. The group of SNPs that slightly deviated from a diagonal straight line in the QQ plot are considered to reflect SNPs with weak genetic effects, and from the plot, it seems that there is not gross inflation of false-positive results derived from genotyping errors.

**Figure 2:**
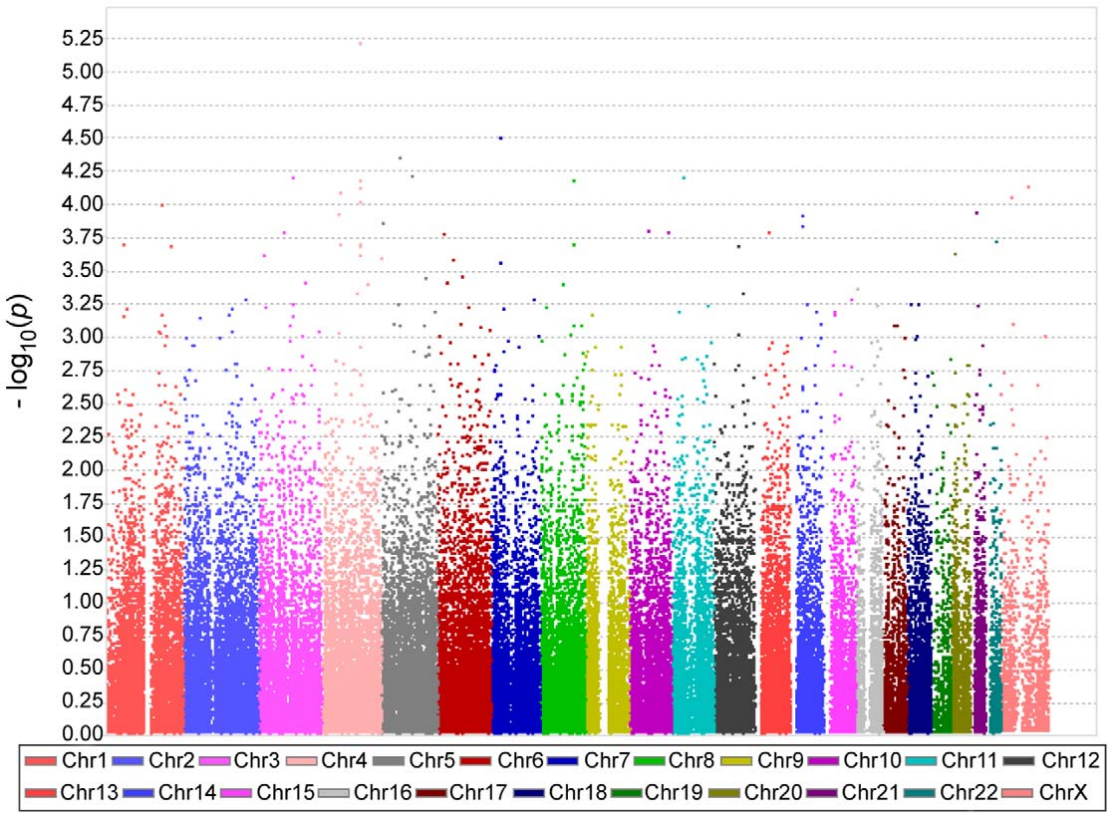
Results of whole genome association scan for Japanese trio samples. A Manhattan plot is shown. SNPs from each chromosome are represented by different colors and ordered by physical positions.

The most significant *p* value was obtained for marker rs2174623 at 4q28.1 (*p* = 6.11×10^−6^), followed by markers rs883955 at Xq24 (*p* = 7.10×10^−6^) and rs10499585 at 7p15.1 (*p* = 3.14×10^−5^). However, no human reference gene was located at these regions. *P* values for the TDT analyses of schizophrenia trios for all 97,963 SNPs are shown in a Manhattan plot (Figure 2).

### Stage II: Replication in Japanese case-control samples

We selected 1,632 SNPs from the first-stage screening [1,159 SNPs of *p*<0.01, and 473 SNPs of *p*<0.05 located in previously reported linkage regions [8], [9]]. In the second-stage analysis, we have taken advantage of an affordable multiplex genotyping platform (Illumina Bead Array). The second sample consisted of 506 patients with schizophrenia and 506 age- and sex-matched controls of Japanese origin. Control subjects were recruited from hospital staff and volunteers who had no family history of psychoses. They showed no current or past evidence of psychoses, during brief interviews by expert psychiatrists. In this experiment, 120 SNPs have dropped owing to the low designability of target SNPs and 40 SNPs could not be genotyped. Accordingly, 1,472 SNPs were successfully genotyped in an independent Japanese case-control sample. Sixty-nine SNPs located on autosomes and 17 SNPs located on chromosome X showed a nominal significance of *p*<0.05 (Tables S1, S2). It may be reasonable not to declare a compelling association regarding these SNPs because of multiple testing, therefore, the interpretation of data must be made with caution. The top 20 SNPs are listed in Table 1. The most significant *p* value in the second-stage analysis was obtained for the SNP in the *ELAVL2* [(embryonic lethal, abnormal vision, Drosophila)-like 2] gene located on 9p21.3 (*p* = 0.00087).

**Table 1:** The top 20 signals in the two-stage association analyses.

RANK	SNP ID	Position	*p* value		Gene	HWE *p* value	
			1st stage	2nd stage		Control	Case
1	rs10491817	9p21.3	0.00649	0.00087	*ELAVL2*	0.576	0.879
2	rs10507559	13q14.2	0.00933	0.00094		0.498	0.382
3	rs9296021	6p21.32	0.01030	0.00158	*C6orf10*	0.240	0.035
4	rs10497106	2q23.3	0.00091	0.00181	*FMNL2*	0.159	0.280
5	rs1899264	2p12	0.02010	0.00240		0.679	0.972
6	rs950651	5p15.32	0.00014	0.00299		0.713	0.256
7	rs1449531	3p24.3	0.00024	0.00299		0.439	0.531
8	rs7970954	12p12.1	0.00511	0.00329	*IFLTD1*	0.900	0.021
9	rs488212	11q22.3	0.01590	0.00386		0.310	0.658
10	rs7695870	4q32.1	0.00953	0.00419	*GRIA2*	0.843	0.975
11	rs660647	11q22.3	0.01510	0.00530		0.345	0.801
12	rs10517668	4q32.1	0.00376	0.00549		0.875	0.682
13	rs2289965	11p15.1	0.00286	0.00678	*IGSF22*	0.273	0.711
14	rs2292101	3p25.2	0.01140	0.00679	*PPARG*	0.817	0.550
15	rs2235394	6p24.1	0.01110	0.00697	*C6orf105*	0.306	0.556
16	rs10496761	2q22.1	0.03250	0.00912		0.099	0.678
17	rs1048076	6p21.1	0.00225	0.00945	*ENPP4*	0.974	0.876
18	rs348116	5p15.2	0.00270	0.00952		0.207	0.918
19	rs10505845	12p12.2	0.00838	0.01010		0.392	0.868
20	rs3106653	2q24.1	0.00465	0.01194	*KCNJ3*	0.710	0.199

Rank is ordered according to the results of the second-stage analysis.

SNP information is listed based on UCSC Feb. 2009 (http://genome.ucsc.edu/).

*P* values of the 1^st^ stage are calculated by Transmission Disequilibrium Test (TDT).

*P* values of the 2^nd^ stage are calculated by Fisher's exact test.

HWE: Hardy Weinberg Equilibrium. Departures from the assumption of HWE are evaluated based on the data from the 2^nd^ stage case-control samples.

### Stage III: Replication in Chinese family samples collected by NIMH

Japanese and Chinese are genetically close, but apparently different populations. In addition, the gene-based approach provides more information than single-SNP analysis, because a high-density mapping could capture the potential risk-conferring variations, which is difficult by examining sparse-density SNPs on the GeneChip.

Accordingly, to confirm association signals in a gene-based manner, we performed a follow-up study of the *ELAVL2* gene whose SNP showed the most compelling association in the case-control study using Japanese population, by densely genotyping 293 pedigree samples (284 quad and 9 trio samples, consisting of 1,163 family members) from Chinese population. We analyzed 56 tagSNPs located in and around the *ELAVL2* gene. This gene has not been reported to be genetically associated with schizophrenia to date.

**Figure 3:**
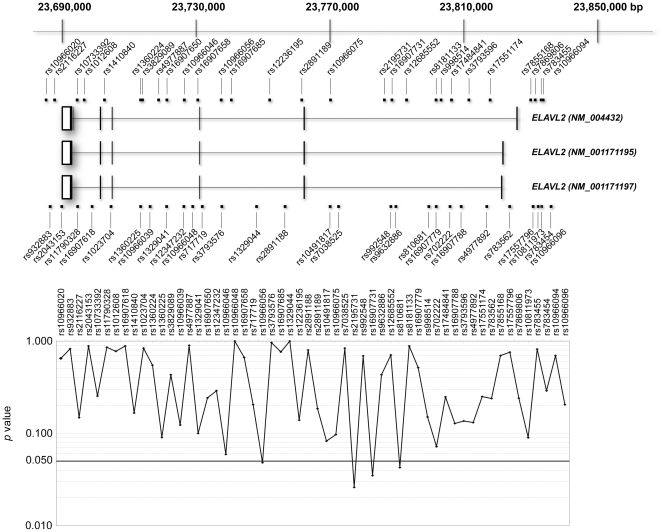
Genomic structure of *ELAVL2* and gene-centric association analysis. Genomic structure and locations of genetic markers in and around *ELAVL2* are shown, with chromosomal positions according to the human genome database (http://genome.ucsc.edu/) on the top. Exons of the gene are denoted by boxes. The negative logarithm of *p* value for association is plotted as a function of chromosomal positions of SNP markers. Bold line indicates *p* value of <0.05.

As shown in Figure 3, single marker analysis in the third set showed a nominally significant association with four SNPs on the gene (lowest *p* = 0.026). Three SNPs are clustered in the intron 1 of the gene. They lose significance when conservative Bonferroni's correction was applied. The transmitted/non-transmitted and overrepresentation/underrepresentation relationship of the allele revealed consistent risk of the minor C allele of the initial marker SNP (rs10491817) in each stage sample. The significant SNPs on the *ELAVL2* gene showed no deviation from Hardy-Weinberg disequilibrium (based on the data from independent parents in the Chinese sample set).

### Quantitative RT-PCR in postmortem brains from schizophrenia

The identification of *ELAVL2* as a susceptibility gene for schizophrenia in both Japanese and Chinese cohorts led us to examine whether the expression levels of the gene are altered in the postmortem brains of patients with schizophrenia. In addition, the accumulating lines of evidence show that schizophrenia and bipolar disorder partly share common susceptibility genes or genetic pathways. We performed real-time quantitative RT-PCR assays for mRNA levels of the gene in the dorsolateral prefrontal cortex (DLPFC: Brodmann's area 46) of schizophrenia, bipolar disorder and control brains.

**Figure 4:**
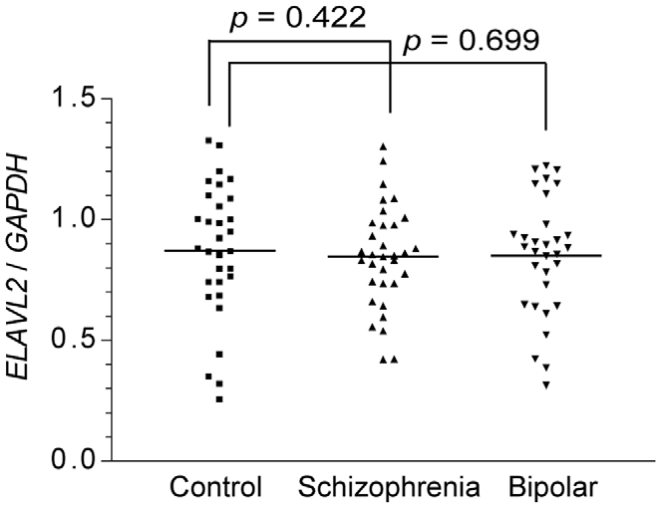
Quantitative RT-PCR in postmortem brains. Rectangles and triangles represent individuals. The horizontal bars delineate the mean of each group. The expression levels of *ELAVL2* were not significantly different among postmortem brains from schizophrenics, bipolar disorder patients and controls in the dorsolateral prefrontal cortex.

However, the experiments showed that the expression levels of *ELAVL2* were not different among brains from schizophrenics, bipolar disorder patients and control subjects (Figure 4). We did not examine the allele-specific expression levels of the transcript because the minor allele (C) frequency is very low (0.056) in Caucasian (from which the postmortem brains are derived) according to the HapMap database (http://hapmap.ncbi.nlm.nih.gov/).

## Discussion

We performed a GWAS, a follow-up replication study and a gene-centric dense mapping to identify susceptibility genes and risk variants for schizophrenia in Japanese and Chinese populations. A novel candidate gene has emerged from our staged association analyses.

ELAVL2 [(embryonic lethal, abnormal vision, Drosophila)-like 2], also known as Hu antigen B (HuB) or Hel-N1, belongs to the RNA-binding Hu (Elavl) protein family. In mammals, the family consists of four highly conserved members that include the ubiquitously expressed ELAVL1 (HuA, HuR) and the neuronal-specific ELAVL2 (HuB, Hel- N1), ELAVL3 (HuC, ple21) and ELAVL4 (HuD) [10], [11], [12], [13], [14]. Neuronal Elavl proteins (nELAVL; ELAVL2, ELAVL3 and ELAVL4) have been identified as splicing regulators in neuron-like cells, and are likely to exert critical posttranscriptional control as key inducers of programmed neuronal differentiation and function in the mammalian nervous system [15], [16]. Given their role in neuronal differentiation and plasticity, nELAVL proteins including ELAVL2 could be potential candidates for neurodegenerative and psychiatric diseases. Indeed, recent studies implicate *ELAVL4* as a Parkinson's disease susceptibility gene [17], [18]. Noteworthy, the altered expression of *GAP43*, one of known targets of ELAVL4, is reported in the frontal cortices and the hippocampus of patients with schizophrenia [19], [20], [21], [22], [23]. Although their relevance to schizophrenia pathogenesis is still awaiting clarification, they are worthy of further investigation.

In this work, we have attempted to minimize several limitations often plaguing association studies in psychiatry. Firstly, to minimize population stratification and genetic heterogeneity, we have focused on trio samples of Japanese descent at the first stage. Secondly, we conducted a replication study for significant SNPs using case-control samples from the same Japanese population but an independent sample set at the second stage. Finally, to confirm the association in an ethnically close but different population, we analyzed patient-parents trios/quads of Chinese descent at the third stage. In this stage, to achieve greater coverage of genetic variations for the survived gene, we performed gene-centric analysis by selecting 56 tag SNPs throughout the entire region of the gene (150 kb).

However, several limitations must be considered. We have not achieved sufficient SNP coverage in the first stage of this study, and the number of samples is modest to detect small to medium effect of a disease-associated gene at genome-wide significance level. When Bonferroni's correction was applied, the significant *p* value was 5.10×10^−7^ for multiple tests of 97,963 SNPs ( = 0.05/97,963). The most significant *p* value in the first stage analysis (*p* = 6.11×10^−6^) loses significance after conservative correction. In addition, a small number of genuine causal variants will be buried within a larger number of SNPs with nominal associations. Therefore, the current study will require a follow-up analysis to distinguish the small number of genuine causal variants from the high proportion of SNPs with false-positive associations. The *ELAVL2* gene showed a nominal significance at each stage. However, the initial marker SNP in Japanese population (rs10491817) was not significant in Chinese population (*p* = 0.082). This may reflect the allelic heterogeneity of the gene, because the significant markers in both populations were not in tight linkage disequilibrium (Figures S1, S2). In this context, the gene warrants further investigation.

Recently, Ikeda and his colleagues reported the first GWAS for schizophrenia in Japanese population [1]. In the study, the strongest associations were observed at rs12218361 mapped near the 3′ end of the *OAT* (a gene for ornithine aminotransferase) on 10q26.13 and rs11895771 mapped within the *SULT6B1* (a gene for sulfotransferase family, cytosolic, 6B, member 1) on 2p22.2 (*p* = 7.2×10^−8^ and *p* = 6.2×10^−6^, respectively). No significant associations for those genes were observed in this study. However, it is noteworthy that the two Japanese studies gave the same gene of nominal significance, *C6orf105* (Table 1). This putative gene is reported as a candidate for non-syndromic oral clefts [24], but its exact function is unknown.

Recent studies show suggestive evidence of association of multiple GABA-related genes with schizophrenia [25], [26]. One of the benefits obtained from GWAS is that we can examine whether a subset of genes categorized into some signaling pathway are involved in the pathogeneses of disease, beyond single genes [27]. We pursued this issue using the first-stage GWAS dataset and unveiled the accumulation of association signals from genes of GABAergic pathways in schizophrenia. Association signature on GABA-related loci was identified across several human chromosomes, which is particularly highlighted on chromosome 5q34 (Figure S3). However, most of the SNPs in and around GABA-related genes associated with schizophrenia in our first-stage dataset were not confirmed in the second-stage samples. Only rs10515831, which lies 90 kb downstream of *GABRB2*, 47 kb upstream of *GABRA6* and 209 kb upstream of *GABRA1* on 5q34, showed a nominal significant association with the disease in the second-stage analysis (*p* = 0.033). This may be due to weak genetic contributions of these genes in Japanese, suggesting the necessity of a much larger number of second-stage samples.

In summary, we provided a suggestive evidence for the contribution of *ELAVL2* to the pathogenesis of schizophrenia, in both Japanese and Chinese populations. This prioritized gene deserves further evaluation to improve the understanding of schizophrenia genetics.

## Materials and Methods

### Samples

A three-stage analysis was performed by using two independent Japanese cohorts and an ethnically close Chinese population. In the first stage, 120 patient-parents trio samples from Japanese schizophrenia pedigrees (360 members) were analyzed. In the second stage, case–control samples consisted of 1,012 unrelated individuals (506 schizophrenia patients, mean age 49.2±13.0 years; 506 age- and sex-matched controls, mean age 49.2±13.0 years). In the third stage, Chinese sample consisted of 293 pedigrees (1,163 subjects: nine trios and 284 quads) collected by the NIMH initiative (http://nimhgenetics.org/). For the Japanese samples, all the subjects resided in central Japan. Consensual diagnoses were made by at least two experienced psychiatrists according to DSM-IV criteria. Written informed consent was obtained from all the participants, after the provision and an explanation of study protocols and purposes. Our case samples in the current study consist of all such patients with schizophrenia who are in a remission/stable chronic state and possess the ability to agree to join the research. This study was approved by the Ethics Committee of RIKEN, and conducted according to the principles expressed in the Declaration of Helsinki.

### First-stage analysis

The first-stage GWAS was performed using Affymetrix GeneChip Mapping 100 K microarrays (Affymetrix, Santa Clara, CA) following the manufacturer's protocol. Genotype data were analyzed with the GeneSpring GT (Varia) 2.0 software package developed by Agilent Technologies (Santa Clara, CA). Transmission disequilibrium test was performed using the R program (http://www.r-project.org). We set a liberal first-stage significance level to increase the potential to detect associated genes with small effects in the subsequent stage analyses: (i) *p* value<0.01, and (ii) *p* value<0.05 when SNPs are located in candidate chromosomal regions detected in the meta-analysis of schizophrenia linkage studies [9] or in the reported linkage regions of Japanese population [8]. We used the Haploview 4.2 to create a Manhattan plot of *p* values from GWAS study (http://www.broadinstitute.org/haploview). A QQ plot of *p* values from GWAS was created using R scripts provided by Diabetes Genetics Initiative (http://www.broadinstitute.org/science/projects/diabetes-genetics-initiative/plotting-genome-wide-association-results). The data obtained in this study have been deposited into the NCBI's Gene Expression Omnibus [28] and are accessible through GEO Series accession number GSE27923 (http://www.ncbi.nlm.nih.gov/geo/query/acc.cgi?acc=GSE27923).

### Second-stage analysis

In the second stage, genotyping was performed using Illumina (San Diego, CA), through the use of their Integrated BeadArray System. We supplied Illumina with 96-well barcoded DNA microtiter plates containing 1,012 samples of DNA (4 mg each) quantified with Pico Green to be 100 ng/ml. Assay quality was as follows: sample success rate of 100%, locus success rate of 97.40%, genotype call rate of 99.98%, reproducibility of 99.997% and genotyping concordance of 99.93%. These results indicate that genotyping was highly accurate and reproducible in this study. Statistical analysis of allelic association was performed using the R program.

### Gene-centric association study

In a gene-centric association study, SNP genotyping was performed using the TaqMan system (Applied Biosystems, Foster City, CA) following the manufacturer's recommendation. PCR was performed using an ABI 9700 thermocycler, and fluorescent signals were analyzed on an ABI 7900HT Fast real-time PCR System using Sequence Detection Software (SDS) v2.3 (Applied Biosystems). TagSNPs were selected using the ldSelect software (http://droog.gs.washington.edu/ldSelect.html) based on their *r^2^* values of 0.8 as a cut-off point to capture genotype information. We genotyped 56 tagSNPs located in and around the *ELAVL2* gene in the stage III. The genetic association was evaluated using the Family-Based Association Test (FBAT) program (v2.0.3, http://www.biostat.harvard.edu/~fbat/).

### Brain tissues and quantitative RT-PCR

RNA from the dorsolateral prefrontal cortex (Brodmann's area 46) was obtained from the Stanley Medical Research Institute (http://sncid.stanleyresearch.org/) [29], [30]. Brain samples were taken from 35 schizophrenics [26 males, 9 females; mean ± SD age, 42.6±8.5 years; postmortem interval (PMI), 31.4±15.5 h; brain pH, 6.5±0.2], 35 bipolar disorder patients (17 males, 18 females; mean ± SD age, 45.3±10.5 years; PMI, 37.9±18.3 h; brain pH, 6.4±0.3), and 35 controls (26 males, 9 females; mean ± SD age, 44.2±7.6 years; PMI, 29.4±12.9 h; brain pH, 6.6±0.3). Diagnoses were made in accordance with DSM-IV criteria. There were no significant demographic differences between the schizophrenia, bipolar and control brains, in terms of age, postmortem interval and sample pH. All the patients with schizophrenia were administered with anti-psychotics. Quantitative RT-PCR analysis was conducted using an ABI7900HT Fast Real-Time PCR System (Applied Biosystems). TaqMan probes and primers for *ELAVL2* and *GAPDH* (an internal control) were Assay-on-Demand™ or Assay-by-Design™ gene expression products (Applied Biosystems). All the quantitative RT-PCR reactions were performed in triplicate, based on a standard curve method. The Mann-Whitney *U* test (two–tailed) was used to evaluate significant changes in target gene expression levels.

## Supporting Information

Table S1 **Replication in Japanese case-control samples (autosomes).** SNP information is based on the UCSC database (http://genome.ucsc.edu/). *P* values of the 1st stage analysis are calculated by Transmission Disequilibrium Test (TDT). *P* values of the 2nd stage analysis are calculated by Fisher's exact test. HWE: Hardy Weinberg Equilibrium. Departures from the assumption of HWE are evaluated based on the data from the 2nd stage case-control samples.
(XLS)

Table S2 **Replication in Japanese case-control samples (chromosome X).** SNP information is based on the UCSC database (http://genome.ucsc.edu/). *P* values of the 1st stage analysis are calculated by Transmission Disequilibrium Test (TDT). *P* values of the 2nd stage analysis are calculated by Fisher's exact test.
(XLS)

Figure S1 **Linkage disequilibrium between markers in Chinese population.** Linkage disequilibrium (LD) between markers constructed by the Haploview program is shown (based on the data from independent parents in the Chinese sample set). The number in each cell represents the LD parameter *r^2^* (×100). Each cell is painted with graduated color relative to the strength of linkage disequilibrium between markers. The rs numbers are SNP I.D. in the NCBI SNP database (http://www.ncbi.nlm.nih.gov/snp). The significant SNPs and the genomic region surrounding these SNPs were shown in red and a red pentagon, respectively.
(TIF)

Figure S2 **Linkage disequilibrium between markers in Japanese population.** Linkage disequilibrium (LD) between markers constructed by the Haploview program using the data from HapMap database is shown (http://hapmap.ncbi.nlm.nih.gov/). The number in each cell represents the LD parameter *r^2^* (×100). Each cell is painted with graduated color relative to the strength of linkage disequilibrium between markers. The rs numbers are SNP I.D. in the NCBI SNP database (http://www.ncbi.nlm.nih.gov/snp). The significant SNPs and the genomic region surrounding these SNPs were shown in red and a red pentagon, respectively.
(TIF)

Figure S3 **Association signals on chromosome 5q GABA_A_ receptor subunit gene cluster.** The chromosome 5q risk locus contains a cluster of GABA_A_ receptor subunit genes, *GABRB2*, *GABRA6*, *GABRA1*, *GABRG2* and *GABRP*. Significant SNPs (*p*<0.05) and the corresponding genes are shown in red.
(TIF)
