# Supplementary figures and images for: Genome-Wide Association Study of Schizophrenia in Japanese Population
Source: PLoS One. 2011 Jun 6;6(6):e20468. doi: 10.1371/journal.pone.0020468 (PMC3108953; doi:10.1371/journal.pone.0020468)

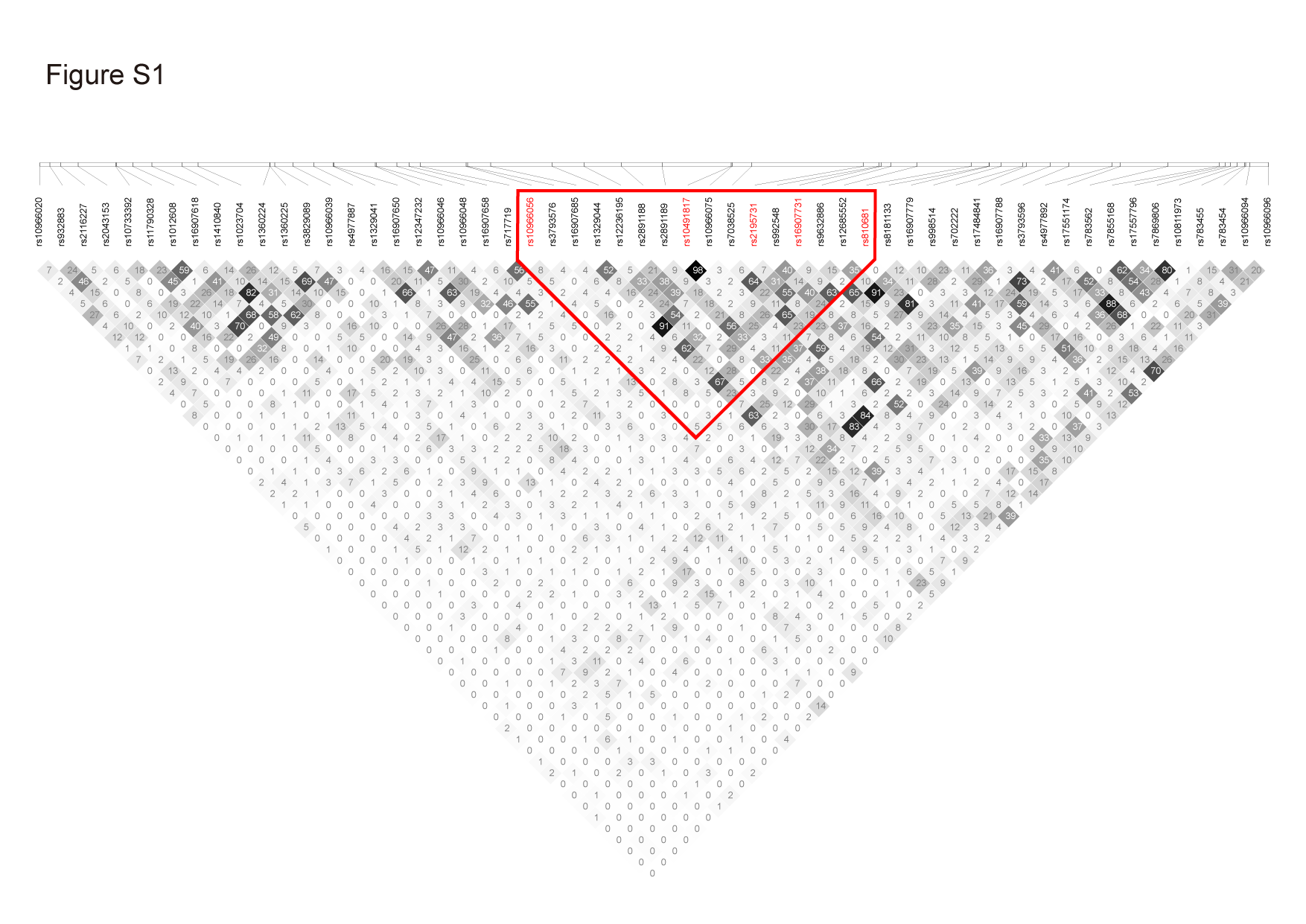

Supplement: Figure S1 — Linkage disequilibrium between markers in Chinese population. Linkage disequilibrium (LD) between markers constructed by the Haploview program is shown (based on the data from independent parents in the Chinese sample set). The number in each cell represents the LD parameter r2 (×100). Each cell is painted with graduated color relative to the strength of linkage disequilibrium between markers. The rs numbers are SNP I.D. in the NCBI SNP database (http://www.ncbi.nlm.nih.gov/snp). The significant SNPs and the genomic region surrounding these SNPs were shown in red and a red pentagon, respectively. (TIF) [file pone.0020468.s003.tif]

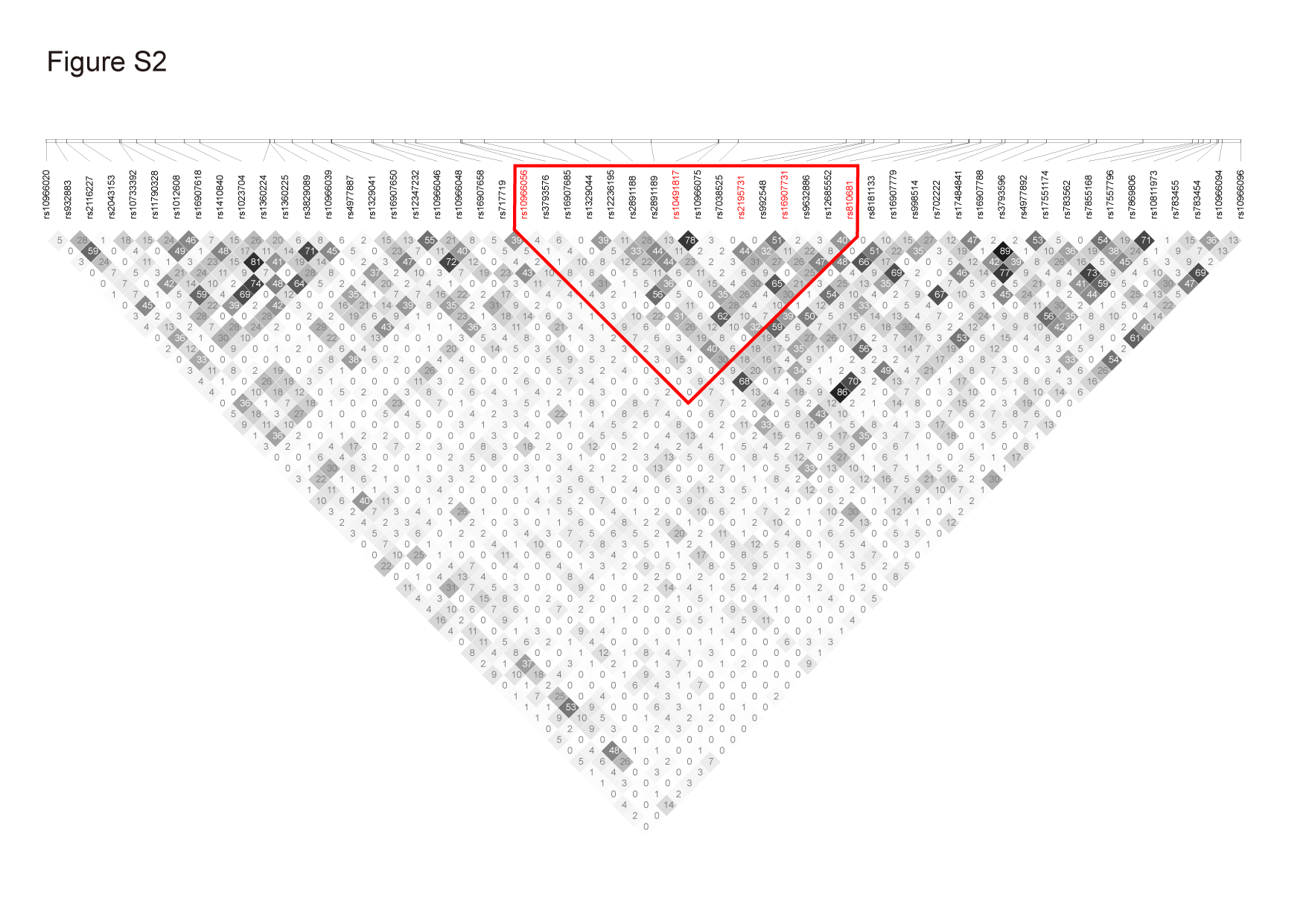

Supplement: Figure S2 — Linkage disequilibrium between markers in Japanese population. Linkage disequilibrium (LD) between markers constructed by the Haploview program using the data from HapMap database is shown (http://hapmap.ncbi.nlm.nih.gov/). The number in each cell represents the LD parameter r2 (×100). Each cell is painted with graduated color relative to the strength of linkage disequilibrium between markers. The rs numbers are SNP I.D. in the NCBI SNP database (http://www.ncbi.nlm.nih.gov/snp). The significant SNPs and the genomic region surrounding these SNPs were shown in red and a red pentagon, respectively. (TIF) [file pone.0020468.s004.tif]

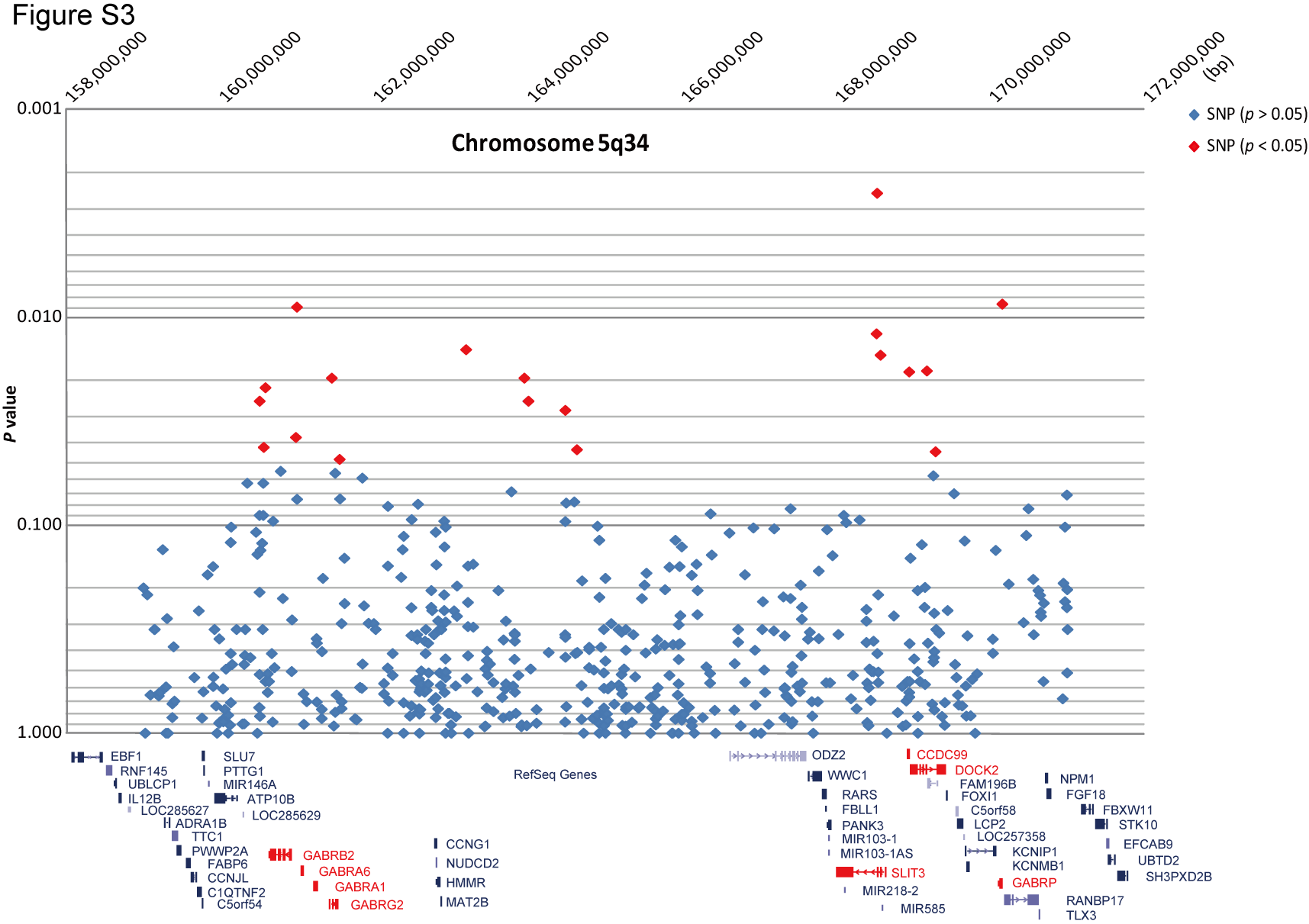

Supplement: Figure S3 — Association signals on chromosome 5q GABAA receptor subunit gene cluster. The chromosome 5q risk locus contains a cluster of GABAA receptor subunit genes, GABRB2, GABRA6, GABRA1, GABRG2 and GABRP. Significant SNPs (p<0.05) and the corresponding genes are shown in red. (TIF) [file pone.0020468.s005.tif]
